# Workforce and Health Care Services for Young Children in Bangladesh

**DOI:** 10.1001/jamanetworkopen.2025.13807

**Published:** 2025-06-05

**Authors:** Md. Monir Hossain Shimul, Md. Kamrul Hossain, Salamat Khandker

**Affiliations:** 1 Department of Public Health, Daffodil International University, Dhaka, Bangladesh; 2 Department of Computer Science and Engineering, Daffodil International University, Dhaka, Bangladesh; 3 Chitkara University Institute of Engineering and Technology, Chitkara University, Punjab, India

## Abstract

**Importance:**

Inequities in pediatric health care services persist in Bangladesh, with critical disparities between public and private hospitals, particularly for children younger than 5 years. These disparities hinder progress toward equitable health care access and quality outcomes.

**Objective:**

To evaluate the availability and quality of health care workforce and service coverage for children younger than 5 years in public and private hospitals in Bangladesh to identify gaps in infrastructure, workforce distribution, and adherence to pediatric care standards.

**Design, Setting, and Participants:**

This cross-sectional study was conducted from October 13, 2023, to May 24, 2024. Data collection included facility assessments, structured questionnaires, and direct observations using World Health Organization (WHO)–adapted tools. The study covered both private and public hospitals across 6 subdistricts in Bangladesh, representing a mix of urban and rural populations. Senior hospital staff and administrators were surveyed. Inclusion criteria were general inpatient and outpatient pediatric care delivered in hospitals. Diagnostic centers and specialized facilities were excluded.

**Exposures:**

Availability of pediatric infrastructure, workforce, essential medications, and adherence to clinical guidelines assessed against WHO standards.

**Main Outcomes and Measures:**

The primary outcomes included the presence of pediatric-specific departments, neonatal care facilities, and trained personnel and adherence to WHO care standards. Data were analyzed using descriptive statistics and χ^2^ tests to highlight disparities.

**Results:**

The study sample included 102 private and 7 public hospitals. A significantly higher proportion of public hospitals compared with private hospitals had pediatric inpatient departments (100% vs 39.2%), newborn wards (100% vs 12.8%), and trained nurses (100% vs 32.4%). Only private hospitals in the sample had neonatal intensive care units (6.9% vs 0%). Pediatricians were available for extended consultations at a greater proportion of public hospitals compared with private hospitals (71.4% vs 26.5%).

**Conclusions and Relevance:**

These findings show substantial disparities in the availability of pediatric health care services between public and private hospitals in Bangladesh. Addressing these disparities through workforce investment and infrastructure improvements is crucial for equitable child health care and better health outcomes.

## Key Points

**Question:**

Are there disparities in health care workforce and service coverage between public and private hospitals in Bangladesh for children younger than 5 years?

**Findings:**

In this cross-sectional study of 102 private and 7 public hospitals, public hospitals had more pediatric inpatient departments, newborn wards, and trained nurses, while private hospitals had more neonatal intensive care unit services. Pediatrician availability for extended consultations was significantly higher in public hospitals.

**Meaning:**

These findings suggest that addressing disparities in the pediatric workforce and infrastructure is essential for equitable health care access and improved child health outcomes.

## Teaser

This cross-sectional study compares the availability and quality of health care workforce and service coverage in public and private hospitals for children younger than 5 years living in Bangladesh.

## Introduction

Child health remains a global priority, yet disparities in health care access and quality persist, especially in low-resource settings such as Bangladesh. Although the Integrated Management of Childhood Illness strategy was introduced in Bangladesh in 1998 to reduce child mortality, crucial gaps in service coverage and the availability of an adequate health care workforce remain unaddressed. Bangladesh is among the 58 countries identified by the World Health Organization (WHO) as experiencing a severe human resources for health crisis, which significantly affects the quality and accessibility of pediatric care.^1,2^

Over the past decade, Bangladesh has made considerable progress in reducing mortality rates among children younger than 5 years, from 31 per 1000 live births in 2015 to 24 per 1000 in 2022. Despite these improvements, inequities in health care services remain prominent, with rural and underserved areas facing acute shortages of trained pediatricians, child health care professionals, and essential facilities.^3,4,5^ Private hospitals dominate Bangladesh’s health care sector but often fail to meet international care standards, further exacerbating disparities.^6,7^ The lack of adherence to clinical guidelines, inadequate infrastructure, and limited human resources highlight the urgent need for systemwide reforms.^8^

The Donabedian model, widely recognized for assessing health care quality through structure, process, and outcomes, provides valuable insight into the multidimensional aspects of health care systems.^9^ However, in this study, we primarily adopted the WHO framework for quality care, which emphasizes evidence-based standards for pediatric health care delivery, focusing on service accessibility, workforce adequacy, and resource availability to improve child health outcomes. Both frameworks underscore the importance of adequate staffing, adherence to clinical guidelines, and the provision of essential drugs and equipment as crucial components of high-quality pediatric care.^10,11,12^

In this study, we evaluated pediatric health care services and workforce availability in public and private hospitals across Bangladesh, addressing a crucial knowledge gap. We hypothesized that disparities may exist between the 2 sectors in infrastructure, workforce distribution, and adherence to pediatric care standards. Using the WHO framework, we aimed to identify gaps in service quality and workforce deployment to offer actionable recommendations for improvement. By generating evidence-based insights, we hope to inform targeted interventions to strengthen Bangladesh’s human resources for health strategy, align health care practices with global standards, and improve equitable access to quality pediatric care.

## Methods

### Study Design

This cross-sectional study assessed the availability of health care workforce and service coverage for children younger than 5 years in 6 subdistricts of Bangladesh. The study was conducted from October 13, 2023, to May 24, 2024, and incorporated both public and private hospitals to provide a comprehensive perspective on pediatric health care. The study received ethical approval from the research ethics committee of the Faculty of Health and Life Sciences, Daffodil International University. Before data collection, senior hospital staff and administrators were briefed on the study’s objectives and procedures. Verbal informed consent was obtained, ensuring that participants understood their rights, including the option to withdraw at any time without consequences. No patient-identifiable data were collected, and the study complied with institutional ethical board standards and the Declaration of Helsinki on medical research.^13^ We followed the Strengthening the Reporting of Observational Studies in Epidemiology (STROBE) reporting guideline.

### Study Area and Population

Bangladesh consists of 495 administrative units called subdistricts or upazilas. We considered each upazila as a cluster. From these 495 clusters, 6 were randomly selected as our study area. Among the selected clusters, Dhamrai and Savar were from Dhaka District, Kapasia from Gazipur District, Manikganj Sadar and Saturia from Manikganj District, and Nangalkot from Cumilla District. The study areas represent a balanced mix of urban and rural populations, with a combined population of 1 994 293 and a total surface area of 1393.18 km^2^.

### Sample Size and Sampling Technique

In 2019, there were 255 public hospitals and 5054 private hospitals and clinics in Bangladesh,^14^ making the total population (N) in the study 5309. For the finite population, the sample size (n) was calculated using the following equation: n = N / (1 + Ne2) = 108.5 ≈ 109, where N = 5309 and the margin of error (e) = 9.5%. To ensure coverage of all types of hospitals (public and private), we collected data from all units within a selected cluster to reach the required sample size of 109 hospitals.

### Inclusion and Exclusion Criteria

The study included hospitals registered with the Directorate General of Health Services that offer general inpatient and outpatient services, tertiary-level institutions, district hospitals, medical colleges, and pediatric care facilities. Exclusion criteria eliminated hospitals not registered with the Directorate General of Health Services, diagnostic centers without inpatient services, community clinics, Union Health Centers, nongovernmental organization–run clinics, and specialized hospitals focusing on diseases or age groups other than pediatrics.

### Respondents and Data Collection Tools

Respondents were senior staff members and administrators of respected hospitals. Data were collected using adapted WHO Hospital Assessment Tools and Standards and a structured questionnaire.^15^ These tools covered hospital infrastructure, equipment, laboratory services, essential drugs, neonatal care, and pediatric workforce. The WHO Hospital Assessment Tools and Standards, which we fully followed in our study assessment, ensure alignment with established patient treatment quality standards, such as the Integrated Management of Childhood Illness, Emergency Triage Assessment and Treatment, and standard operating procedures.

### Data Collection and Quality Control

Data collection involved face-to-face interviews, facility walkthroughs, and direct observation of clinical practices by assessor pediatricians. To mitigate potential biases, particularly the differences observed between urban hospitals (which often have more comprehensive record keeping and readily available staff) and rural facilities (which may face challenges, eg, intermittent power supply and limited staffing), we standardized our data collection process. For instances in which direct patient observations were unavailable, simulated cases were used, and all findings were rigorously cross-verified with hospital records and management information system reports.

Eight assessors, including MBBS physicians (of whom M.M.H.S. was one), MPH students with child health research experience, and practicing pediatricians with postgraduate degrees, were organized into 4 teams. Each team, which comprised 1 pediatrician and 1 public health expert, conducted 2 hospital visits between January 3 and March 28, 2024, ensuring comprehensive assessments across various settings. To further address potential data collection biases, all assessors underwent extensive online training and participated in field practice sessions designed to harmonize data collection techniques across both rural and urban settings.

Hospitals were evaluated against WHO quality standards, with each facility receiving a score ranging from 1 to 5 based on key variables such as pediatric staff availability, essential medications, neonatal care facilities, and patient treatment quality. A score of 5 indicated full compliance, while lower scores signified gaps in quality or resources. This structured approach, combined with rigorous quality control measures, aimed to minimize methodological biases and ensure consistency across the study.

### Statistical Analysis

The data were verified and analyzed using SPSS, version 23.0 (IBM Corp). Descriptive statistics were used to summarize findings, while χ^2^ tests were performed to check the status and its associated factors between private and public hospitals. Results were tabulated to highlight disparities in service readiness and adherence to standards, with the aim to guide improvements in health care services. The threshold for significance was set at *P* < .05.

## Results

A total of 109 hospitals (102 private and 7 public) among the 6 surveyed subdistricts in Bangladesh were studied. Savar had the highest number of private hospitals (42 [41.2%]), followed by Manikganj Sadar (34 [33.3%]); Saturia had the lowest number of private hospitals (2 [2.0%]) (eTable 1 in Supplement 1). The public hospitals were distributed across the study area, with Manikganj Sadar having 2 (28.6%) (1 being the District Sadar Hospital and 1 a government medical college hospital). The remaining subdistricts (Savar, Dhamrai, Saturia, Kapasia, and Nangalkot) each had 1 public hospital (14.3%), designated as the Upazila Health Complex. Specialized health care services for children were notably scarce, with only 1 specialized child hospital located in Savar. All hospitals across the study area provided 24-hour services with 24-hour physician availability.

### Child Health Infrastructure and Support System

Public hospitals provided more services and infrastructure compared with private hospitals in crucial areas, with significantly higher proportions having separate pediatric inpatient departments (100% vs 39.2%; *P* = .001), newborn wards (100% vs 12.8%; *P* < .001), and trained nurses for child care (100% vs 32.4%, *P* < .001) available (Table 1). They also led in pediatric surgery facilities (71.4% vs 15.7%; *P* < .001), employment of pediatricians (71.4% vs 10.8%; *P* < .001), and pediatric surgeons (57.1% vs 7.8%; *P* < .001). Emergency services were significantly better in public hospitals, with 24/7 services (100% vs 33.3%; *P* < .001) and a functioning triage system (42.9% vs 12.8%; *P* = .02). However, neonatal intensive care unit facilities were not available in public hospitals (0%), while 6.9% of private hospitals provided them (*P* < .001). Compared with public hospitals, private hospitals were less likely to provide complaint mechanisms (17.7% vs 100%; *P* < .001); in addition, a smaller percentage of private hospitals provided drug and vaccine storage facilities (83.3% vs 100%; *P* = .07), although the difference was not statistically significant. Both types of hospitals provided basic utilities, such as electricity, backup power, fuel, and water, but public hospitals were better equipped to deliver comprehensive child health care services.

**Table 1.** Availability of Child Health Infrastructure and Support Systems in Private and Public Hospitals in Bangladesh

Variable	Hospitals, No. (%)		*P* value
	Private (n = 102)	Public (n = 7)	
Separate pediatric outpatient department	88 (86.3)	7 (100)	.41
Separate pediatric inpatient department	40 (39.2)	7 (100)	.001
Separate ward for admitting newborns	13 (12.8)	7 (100)	<.001
Hospital has a neonatal intensive care unit	7 (6.9)	0	<.001
Pediatric surgical facilities	16 (15.7)	5 (71.4)	<.001
Employment of any pediatrician	11 (10.9)	5 (71.4)	<.001
Employment of any pediatric surgeon	8 (7.8)	4 (57.1)	<.001
Employment of trained nurse for child care	33 (32.4)	7 (100)	<.001
24/7 Emergency services for children	34 (33.3)	7 (100)	<.001
Functioning triage system for emergency services for children	13 (12.8)	3 (42.9)	.02
Safe drinking water	90 (88.2)	7 (100)	.41
Colored bins for waste segregation and disposal	82 (80.4)	7 (100)	.08
Functioning refrigerator for drugs or vaccines	85 (83.3)	7 (100)	.07
Complaints box on the hospital premises or a formal way for patients to communicate with the hospital	18 (17.7)	7 (100)	<.001

### Hospital Equipment, Essential Drugs, and Laboratory Supports

Public hospitals were significantly better equipped compared with private hospitals for neonatal emergencies, with higher proportions of available incubators and warmers (57.1% vs 14.7%; *P* = .01). Public hospitals also had greater availability of pediatric-specific supplies, such as infusion sets (100% vs 39.2%, *P* < .001), pediatric-sized butterfly needles and/or cannulas (100% vs 39.2%; *P* < .001), and nasogastric tubes (71.4%% vs 12.8%; *P* < .001). Both types of hospitals provided weighing scales in inpatient departments and suction equipment.

In terms of essential drugs (Table 2), a higher proportion of public hospitals compared with private hospitals had consistently greater, though not statistically significant, availability of important medications, including intravenous glucose, diazepam, phenobarbital, ampicillin, amoxicillin, and epinephrine. Both public and private hospitals reliably stocked other key medications, such as normal intravenous saline, intravenous cholera saline, oral rehydration solutions, corticosteroids, injectable ceftriaxone, injectable vitamin K, amoxicillin syrup, and nebulizer solutions.

**Table 2.** Availability of Hospital Equipment, Essential Drugs, and Laboratory Support in Private and Public Hospitals in Bangladesh

Variable	Hospitals, No. (%)		*P* value
	Private (n = 102)	Public (n = 7)	
**Availability of equipment**			
Filled oxygen cylinder	88 (86.3)	7 (100)	.41
Flow meters for oxygen cylinder	88 (86.3)	7 (100)	.41
Incubators for emergency neonatal care	15 (14.7)	4 (57.1)	.01
Warmers for emergency neonatal care	15 (14.7)	4 (57.1)	.01
Measuring board for length and height	89 (87.3)	7 (100)	.41
Infusion sets for pediatric use	40 (39.2)	7 (100)	<.001
Butterfly needles and/or cannulas, pediatric size	40 (39.2)	7 (100)	<.001
Nasogastric tubes, pediatric size	13 (12.8)	5 (71.4)	<.001
Equipment cleaned regularly and sanitized	86 (84.3)	6 (85.7)	>.99
Equipment kept in good working order	82 (80.4)	5 (71.4)	.62
**Availability of essential drugs**			
Glucose 10% IV	75 (73.5)	7 (100)	.41
First-line anticonvulsant: diazepam; paraldehyde IM, IV, and suppository	86 (84.3)	7 (100)	.41
Phenobarbital IM and IV	86 (84.3)	6 (85.7)	>.99
Injectable ampicillin	80 (78.4)	6 (85.7)	>.99
Injectable amoxicillin	89 (87.3)	7 (100)	.41
Injectable gentamicin	92 (90.2)	6 (85.7)	.62
Injectable epinephrine (adrenaline)	75 (73.5)	7 (100)	.41
Co-trimoxazole tablet	80 (78.4)	6 (85.7)	>.99
Zinc syrup, tablet	90 (88.2)	7 (100)	.41
**Availability of laboratory support**			
Essential tests 24/7	60 (58.8)	4 (57.1)	.89
Supplies for hematocrit (PCV)	86 (84.3)	5 (71.4)	.19
Supplies for bilirubin	69 (67.7)	5 (71.4)	.73
Supplies for electrolytes	28 (27.5)	3 (42.9)	.24
Supplies for malaria microscopy	82 (80.4)	6 (85.7)	.61
Blood grouping and cross-matching	96 (94.1)	5 (71.4)	.01
HIV tests	79 (77.5)	6 (85.7)	.46
Tests according to standard operating procedures	86 (84.3)	5 (71.4)	.19
Laboratory safety measures	88 (86.3)	6 (85.7)	.89

Abbreviations: IM, intramuscular; IV, intravenous; PCV, packed cell volume.

For laboratory services, private hospitals excelled over public hospitals in proportions with available blood grouping and cross-matching (94.1% vs 71.4%; *P* = .01) (Table 2). Public hospitals, however, showed better, albeit nonsignificant, availability of important tests such as hematocrit and bilirubin. Both hospital types provided essential laboratory services, including blood glucose and hemoglobin tests, full blood counts, microscopy for blood and urine cells, and access to x-ray equipment.

### Availability and Quality of Neonatal Care

Public hospitals outperformed private hospitals in neonatal care (Table 3), including higher proportions with availability of separate units for seriously ill neonates (42.9% vs 7.8%; *P* = .02) and immunization services (100% vs 3.9%; *P* < .001), which are universally available in public hospitals but limited in private hospitals. Both hospital types promoted early initiation of exclusive breastfeeding, yet gaps persisted between public and private hospitals in the availability of 24/7 trained staff for neonatal resuscitation (57.1% vs 23.5%; *P* = .01). Public hospitals also showed better performance in ensuring hygienic wards (57.1% vs 27.4%) and accurately diagnosing low birth weight newborns (85.7% vs 61.8%).

**Table 3.** Availability and Quality of Neonatal Care in Private and Public Hospitals in Bangladesh

Variable	Adequacy of items, No. (%)		Improvements needed, No. (%)		*P* value
	Private hospitals (n = 102)	Public hospitals (n = 7)	Private hospitals (n = 102)	Public hospitals (n = 7)	
Guidelines for newborn resuscitation are available, and staff are trained to use them	47 (46.1)	3 (42.9)	55 (53.9)	4 (57.1)	>.99
24/7 Staff available to appropriately help a newborn breathe and to perform resuscitation appropriately	24 (23.5)	4 (57.1)	78 (76.5)	3 (42.9)	.01
Clean resuscitation bed with heating and ready-to-use equipment	60 (58.8)	5 (71.4)	42 (41.2)	2 (28.6)	.80
Hygienic and safe ward environment	28 (27.5)	4 (57.1)	74 (72.6)	3 (42.9)	.21
Separate unit or room for critically ill neonates	8 (7.8)	3 (42.9)	94 (92.2)	4 (57.1)	.02
Early exclusive breastfeeding, with skin-to-skin contact promoted	86 (84.3)	6 (85.7)	16 (15.7)	1 (14.3)	>.99
Practices ensuring thermal protection	24 (23.5)	4 (57.1)	78 (76.5)	3 (42.9)	.13
Availability of immunization	4 (3.9)	7 (100)	98 (96.1)	0	<.001
Accurate diagnosis of low birth weight newborns	63 (61.8)	6 (85.7)	39 (38.2)	1 (14.3)	.39
Proper treatment for low birth weight newborns	27 (26.5)	4 (57.1)	75 (73.5)	3 (42.9)	.19
Appropriate feeding for young and low birth weight infants	25 (24.5)	4 (57.1)	77 (75.5)	3 (42.9)	.15

### Quality of Case Management by Illness

For pneumonia, a higher proportion of private hospitals compared with public hospitals had significantly better performance in diagnosing and managing tuberculosis (78.4% vs 28.6%; *P* = .003) (eTable 2 in Supplement 1), highlighting a significant disparity. However, no significant differences were observed in the correct assessment and treatment of pneumonia severity or the administration of oxygen. For diarrhea management, public hospitals significantly outperformed private hospitals in administering appropriate antibiotics only when necessary (57.1% vs 19.6%; *P* = .02). While public hospitals also showed better performance in assessing dehydration (71.4% vs 40.2%) and implementing rehydration plans (57.1% vs 27.5%), these differences were not statistically significant. For malnutrition, public hospitals showed higher adequacy in all evaluated domains, including proper management of dehydration (57.1% vs 33.3%) and electrolyte imbalances (57.1% vs 30.4%), though these differences were not significant.

### Monitoring and Follow-Up

Regarding nutritional assessment, a higher proportion of public hospitals compared with private hospitals adequately assessed all admitted patients (28.6% vs 15.0%), though the difference was not statistically significant (Table 4). Similarly, the use of monitoring charts for individual progress was low in both public and private hospitals (28.6% vs 25.0%). For key risk monitoring, a higher, but nonsignificant, proportion of private hospitals compared with public hospitals had adequately recorded key risk signs by nurses (40.0% vs 28.6%). Reassessment by physicians during working days was more frequent in public compared with private hospitals (57.1% vs 27.5%), as was weekend or holiday review (57.1% vs 27.5%), although neither difference was statistically significant. Reassessment before discharge was better among nurses in private compared with public hospitals (60.0% vs 57.1%), but physician reassessment before discharge was higher in public compared with private hospitals (42.9% vs 17.5%); neither were statistically significant. For discharge notes, private hospitals were more likely to provide detailed notes with follow-up schedules compared with public hospitals (65.0% vs 42.9%), but the difference was not statistically significant.

**Table 4.** Monitoring and Follow-Up of Admitted Pediatric Patients in Private and Public Hospitals in Bangladesh

Variable	Adequacy of items, No. (%)		Improvements needed, No. (%)		*P* value
	Private hospitals (n = 102)	Public hospitals (n = 7)	Private hospitals (n = 102)	Public hospitals (n = 7)	
Nutritional status assessed in all admitted patients	6 (15.0)	2 (28.6)	34 (85.0)	5 (71.4)	.21
Use of chart for monitoring individual progress	10 (25.0)	2 (28.6)	30 (75.0)	5 (71.4)	.84
Key risk signs monitored and recorded by a nurse	16 (40.0)	2 (28.6)	24 (60.0)	5 (71.4)	.42
All patients reassessed daily during working days by a physician	11 (27.5)	4 (57.1)	29 (72.5)	3 (42.9)	.09
Sick children or newly admitted children also reviewed by a physician on weekends and holidays	11 (27.5)	4 (57.1)	29 (72.5)	3 (42.9)	.09
Reassessment of all admitted children by a nurse before discharge	24 (60.0)	4 (57.1)	16 (40.0)	3 (42.9)	.90
Reassessment of all admitted children by a physician before discharge	7 (17.5)	3 (42.9)	33 (82.5)	4 (57.1)	.09
Discharge note explaining the conditions and information about follow-up necessities and schedule	26 (65.0)	3 (42.9)	14 (35.0)	4 (57.1)	.19

### Availability of Pediatricians in Hospitals

A significantly higher proportion of private hospitals provided more pediatrician consultations for 3 or fewer days compared with public hospitals (73.5% vs 28.6%; *P* = .01) (Table 5). Conversely, public hospitals offered significantly more consultations for more than 3 days compared with private hospitals (71.4% vs 26.5%; *P* = .01).

**Table 5.** Association Between Availability of Pediatrician and Type of Hospital

Variable	Hospitals, No. (%)		*P* value
	Private (n = 102)	Public (n = 7)	
Availability of pediatrician consultation			
≤3 d	75 (73.5)	2 (28.6)	.01
>3 d	27 (26.5)	5 (71.4)	
No. of pediatricians			
≤2	88 (86.3)	5 (71.4)	.28
>2	14 (13.7)	2 (28.6)	

Regarding the number of pediatricians, most private hospitals employed 2 or fewer pediatricians, similar to public hospitals (86.3% vs 71.4%). However, having more than 2 pediatricians was more common in public hospitals compared with private hospitals (28.6% vs 13.7%) (Table 5; eTable 3 in Supplement 1).

## Discussion

This cross-sectional study reveals important disparities in pediatric health care services and workforce availability between public and private hospitals in Bangladesh, particularly for children younger than 5 years. The findings highlight several interconnected issues, including a shortage of pediatricians, inadequate hospital infrastructure, poor neonatal care, and deficiencies in clinical case management. These issues are consistent with previous studies conducted both in Bangladesh and other developing countries, which have identified similar challenges in the availability and quality of pediatric health care services.^16,17,18^ These results underscore the need for national health care policies that prioritize equitable resource allocation and the adoption of global best practices, such as adherence to WHO guidelines, to bridge the gap between public and private sectors.^16,17,18^

In the past decade, while some improvements have been made in child care, such as investments in private health care and improvements in public hospital conditions, challenges remain. A previous study emphasized that district hospitals play a crucial role in reducing child mortality,^19^ yet our study revealed that many district-level hospitals, including both public medical colleges and private facilities, lacked essential services such as neonatal intensive care units. The recurring “referred to Dhaka Child Hospital” note in most patient records underscores the limitations faced by all studied hospitals. The insights gained from these observations may guide policy makers to target infrastructural deficits and develop strategies that integrate best practices from both national and international frameworks.^19^

Our study underscores substantial rural-urban disparities in pediatric health care access in Bangladesh in which rural and subdistrict areas remain severely underserved.^20^ Although the number of pediatricians has increased, their distribution is uneven, with private hospitals often lacking pediatricians or employing only 1 specialist. Public hospitals, while better staffed, face a shortage of pediatric surgeons, limiting their ability to deliver comprehensive care. High operational costs in private hospitals and underinvestment in specialized staff exacerbate these disparities. Similar trends are observed in neighboring countries, including India and Nepal, in which rural areas experience higher child morbidity and mortality due to inadequate access to specialized health care.^16,17^ Addressing these inequities requires urgent investment in primary health care services in underserved areas to enhance workforce distribution and infrastructure. Strengthened health care delivery systems are essential to ensure timely and adequate care for children, particularly in regions in which the absence of specialized services continues to jeopardize child health outcomes. Furthermore, while our study’s selection of 6 subdistricts provides critical insights, we acknowledge that this sample may not capture the full diversity of Bangladesh’s socioeconomic landscape. Future research should expand the geographic scope to include a broader range of regions, thereby enhancing equity, diversity, and inclusion considerations in policy formulation.

In terms of neonatal care, public hospitals in Bangladesh performed better than private hospitals in key areas such as neonatal resuscitation and the availability of separate units for critically ill neonates. These results are consistent with findings from similar studies in other low-resource settings, such as Ethiopia in which public hospitals were found to have more comprehensive neonatal care despite similar resource limitations.^21^ The lack of trained staff for neonatal resuscitation in private hospitals underscores a crucial gap in pediatric care. Timely and effective neonatal care is essential to preventing neonatal and infant mortality, which remain substantial health challenges in low-resource settings.^22^

Public hospitals in Bangladesh also had better adherence to clinical guidelines in the management of common childhood illnesses, such as pneumonia, diarrhea, and malnutrition. This finding is in line with those from other low- and middle-income countries in which public hospitals, despite facing resource constraints, are often better equipped to follow evidence-based practices, particularly in the management of infectious diseases.^21,23^ For example, the more judicious use of antibiotics in public hospitals is an essential strategy for combating the growing threat of antibiotic resistance, which is a concern across health care systems worldwide.^24^

Our study also highlights the need for improved monitoring and follow-up systems in both public and private hospitals. Low rates of nutritional assessments and inadequate monitoring of key risk factors were observed in both sectors. Regular monitoring is crucial for preventing and treating chronic conditions in children, particularly those with malnutrition or other long-term health issues. This issue has been identified in several global studies, particularly in low- and middle-income countries in which inadequate monitoring contributes to poor health outcomes for children, especially those with malnutrition or other chronic conditions.^25^

Furthermore, the disparity in pediatric workforce distribution between public and private hospitals remains an important issue. Public hospitals tend to employ more pediatric specialists, which is crucial for ensuring that children receive appropriate care. Global studies have consistently shown that an adequate pediatric workforce is essential for improving child health outcomes and reducing mortality rates.^26^ The findings of this study emphasize the need for targeted interventions to address these workforce disparities, particularly in underserved regions, to improve pediatric care access.

Our findings underscore significant deficiencies in Bangladesh’s pediatric health care system, emphasizing the need for urgent intervention in both public and private sectors. Improving workforce capacity, infrastructure, and adherence to WHO guidelines may reduce child morbidity and mortality, especially in underserved areas. The study calls for further research to explore the barriers to health care delivery and evaluate the long-term impact of interventions, workforce training, and policy reforms.

### Recommendations

On the basis of these findings, we have several recommendations to improve the availability and quality of pediatric health care services in Bangladesh. First, both the public and private sectors should increase investments in pediatric-specific facilities, such as neonatal intensive care units and inpatient departments, to provide comprehensive care for children. Second, more trained pediatricians, pediatric surgeons, and trained nurses are needed in both sectors to ensure that specialized care is available for all children. Third, ensuring that all hospitals, particularly private hospitals, have robust support systems, including safe drinking water and waste management, is crucial for maintaining hygiene and operational efficiency. Fourth, both sectors should ensure a reliable supply of proper equipment, essential drugs, and laboratory services to facilitate timely diagnosis and treatment. Fifth, private hospitals should adopt and adhere to WHO guidelines and standards to improve the quality of care and clinical case management for pediatric emergencies. Finally, structured programs for neonatal and pediatric care should be implemented across both private and public sectors to manage common childhood illnesses and provide nutritional support effectively.

### Strengths and Limitations

Strengths of this study included the use of reliable WHO assessment tools and providing a comprehensive overview. Despite these strengths, this study had several limitations. The study’s cross-sectional design and limited regional focus posed challenges in drawing causal inferences or capturing all health care variations. Additionally, the study’s focus on 6 subdistricts, while providing valuable insights, may not encompass regional disparities across Bangladesh, particularly in remote or underserved areas. The exclusion of nonregistered facilities, nongovernmental organization–run clinics, community clinics, and Union Health Centers restricts the generalizability of the findings, as these institutions play an important role in health care delivery. Further classification or tiering of private hospitals should be established at the government level during hospital authorization processes to facilitate a more comprehensive analysis of health care service disparities and human resource distribution.

## Conclusions

This cross-sectional study reveals notable differences between public and private hospitals in the availability and quality of health care services for children younger than 5 years in Bangladesh. Private hospitals faced a more severe shortage of pediatricians, while public hospitals struggled with essential medical supplies and equipment. This situation was exacerbated by a severe shortage of pediatricians, particularly in private hospitals, and inadequate neonatal care facilities. Despite the presence of some amenities that aid in treating sick children, the overall service quality remained substandard, aligning with findings from previous studies in similar low-resource settings.

## Notes

Supplement 1. **eTable 1.** Clusterwise General Information About Studied Hospitals **eTable 2.** Quality of Case Management by Illness (Pneumonia, Diarrhea, Malnutrition) in Private and Public Hospitals in Bangladesh **eTable 3.** Availability of Pediatricians in Private and Public Hospitals in Bangladesh

Supplement 2.
Data Sharing Statement
