# Supplementary material for: Workforce and Health Care Services for Young Children in Bangladesh
Source: JAMA Netw Open. 2025 Jun 5;8(6):e2513807. doi: 10.1001/jamanetworkopen.2025.13807 (PMC12142442; doi:10.1001/jamanetworkopen.2025.13807)
Supplement: Supplement 1. — eTable 1. Clusterwise General Information About Studied Hospitals eTable 2. Quality of Case Management by Illness (Pneumonia, Diarrhea, Malnutrition) in Private and Public Hospitals in Bangladesh eTable 3. Availability of Pediatricians in Private and Public Hospitals in Bangladesh [file jamanetwopen-e2513807-s001.pdf]

Supplemental Online Content

Shimul MH, Hossain K, Khandker S. Workforce and health care services for young children in Bangladesh. *JAMA Netw Open*. 2025;8(6):e2513807.
doi:10.1001/jamanetworkopen.2025.13807

eTable 1. Clusterwise General Information About Studied Hospitals

eTable 2. Quality of Case Management by Illness (Pneumonia, Diarrhea, Malnutrition) in Private and Public Hospitals in Bangladesh

eTable 3. Availability of Pediatricians in Private and Public Hospitals in Bangladesh

This supplemental material has been provided by the authors to give readers additional information about their work.

© 2025 Shimul MH et al. *JAMA Network Open*.

eTable 1. Clusterwise General Information About Studied Hospitals

Variables	Savar Upazila	Dhamrai Upazila	Manikganj Sadar Upazila	Saturia Upazila	Kapasia Upazila	Nangalkot Upazila	Total
Total Private Hospital	42	12	34	2	8	4	102
Total Public Hospital	1	1	2	1	1	1	7
Specialized Child Hospital	1	0	0	0	0	0	1
24 hours open	43	13	36	3	9	5	109
24 hours doctor availability	43	13	36	3	9	5	109

© 2025 Shimul MH et al. *JAMA Network Open*.

eTable 2. Quality of Case Management by Illness (Pneumonia, Diarrhea, Malnutrition) in Private and Public Hospitals in Bangladesh

Items	Adequacy of items		Improvements needed		P Value
	Private	Public	Private	Public	
**Pneumonia**					
Correct assessment and diagnosis of the severity of pneumonia	64 (62.75%)	4 (57.14%)	38 (37.25%)	3 (42.86%)	.77
Appropriate administration of antibiotics for pneumonia and other respiratory diagnosis	64 (62.75%)	4 (57.14%)	38 (37.25%)	3 (42.86%)	.77
Correct administration of oxygen when necessary	50 (49.02%)	3 (42.86%)	52 (53.98%)	4 (57.14%)	.75
Appropriate diagnosis and management of tuberculosis	80 (78.43%)	2 (28.57%)	22 (21.57%)	5 (71.43%)	.003
**Diarrhea**					
Correct assessment of dehydration	41 (40.20%)	5 (71.43%)	61 (59.80%)	2 (28.57%)	.11
Administration and monitoring of rehydration plans appropriate to the severity of dehydration	28 (27.45%)	4 (57.14%)	74 (72.54%)	3 (42.86%)	.09
Appropriate antibiotics administered only when necessary	20 (19.61%)	4 (57.14%)	82 (80.39%)	3 (42.86%)	.02
Continued feeding given during diarrhoea	81 (79.41%)	5 (71.43%)	21 (20.58%)	2 (28.57%)	.61
**Malnutrition**					
Nutritional status assessed by weight for height correctly, including differential diagnosis	40 (39.22%)	4 (57.14%)	62 (60.78%)	3 (42.86%)	.34
Appropriate management of infection	40 (39.22%)	4 (57.14%)	62 (60.78%)	3 (42.86%)	.34
Appropriate management of electrolyte imbalance and micronutrients	31 (30.39%)	4 (57.14%)	71 (69.61%)	3 (42.86%)	.14
Correct management of dehydration	34 (33.33%)	4 (57.14%)	68 (66.67%)	3 (42.86%)	.20
Hypoglycaemia and hypothermia correctly checked and managed	32 (31.37%)	4 (57.14%)	70 (68.63%)	3 (42.86%)	.16
Correct feeding of severely malnourished children	26 (25.49%)	4 (57.14%)	76 (74.51%)	3 (42.86%)	.06

© 2025 Shimul MH et al. *JAMA Network Open*.

eTable 3. Availability of Pediatricians in Private and Public Hospitals in Bangladesh

Total days of Paediatrician consultation	Number of Private hospitals (n= 102)	Number of Public hospitals (n= 7)
0 day	14 (13.73%)	2 (28.57%)
1 day	24 (23.53%)	0 (0%)
2 days	30 (29.41%)	0 (0%)
3 days	7 (6.86%)	0 (0%)
4 days	12 (11.76%)	1 (14.29%)
5 days	0 (0%)	1 (14.29%)
6 days	11 (10.78%)	2 (28.57%)
7 days	4 (3.92%)	1 (14.29%)
**Total number of attending Paediatricians**	**Number of Private hospitals (n= 102)**	**Number of Public hospitals (n= 7)**
0	14 (13.73%)	2 (28.57%)
1	50 (49.02%)	1 (14.29%)
2	24 (23.53%)	2 (28.57%)
3	6 (5.88%)	0 (0%)
4	6 (5.88%)	0 (0%)
5+	2 (1.96%)	2 (28.57%)

© 2025 Shimul MH et al. *JAMA Network Open*.
