# Supplementary material for: Workforce and Health Care Services for Young Children in Bangladesh
Source: JAMA Netw Open. 2025 Jun 5;8(6):e2513807. doi: 10.1001/jamanetworkopen.2025.13807 (PMC12142442; doi:10.1001/jamanetworkopen.2025.13807)
Supplement: Supplement 2. — Data Sharing Statement [file jamanetwopen-e2513807-s002.pdf]

Data Sharing Statement

Shimul. Workforce and Health Care Services for Young Children in Bangladesh. *JAMA Netw Open*. Published June 05, 2025. doi:10.1001/jamanetworkopen.2025.13807

Data
Data available: Yes
Data types: Data (not involving human participants)
How to access data: shimul2375@gmail.com
When available: With publication

Supporting Documents
Document types: None

Additional Information
Who can access the data: Researchers whose proposed use of the data has been approved
Types of analyses: For a specified purpose
Mechanisms of data availability: Without investigator support
